# Stacked Uncertainties: Understanding Educator Well-Being and Resilience in an Era of Rapid Change

**DOI:** 10.3390/bs16081403

**Published:** 2026-08-16

**Authors:** Breanna C. Lawrence, Linnea Leist

**Affiliations:** Educational Psychology & Leadership, Faculty of Education, University of Victoria, Victoria, BC V8P 5C2, Canada

**Keywords:** resilience, educator well-being, social ecological systems, coping, uncertainty, coping, educational change

## Abstract

Educators are working within increasingly complex environments characterized by rapid change and uncertainty, where future conditions, expectations, and appropriate responses are often ambiguous or difficult to anticipate. Technological innovation, political and social polarization, climate-related concerns, shifting policy landscapes, workforce pressures, and the lasting effects of public health disruptions each introduce distinct yet interconnected forms of uncertainty. While these domains of uncertainty have generated substantial bodies of scholarship, less attention has been paid to how multiple forms of uncertainty converge within educators’ professional lives. This essay introduces the concept of *stacked uncertainties* as a lens for understanding how overlapping uncertainties accumulate, interact, and amplify across interconnected technological, socio-political, ecological, organizational, and policy systems. From this perspective, multiple uncertainties compete for the same personal, relational, organizational, and material resources that support educator well-being and resilience. Resilience is understood as a dynamic process shaped by access to adaptive resources across ecological systems. Applying the concept of stacked uncertainties has practical implications for educational leadership, policy, professional learning, and initiatives aimed at strengthening conditions that support educator well-being and resilience amid ongoing complexity and change.

## 1. Introduction

Educator resilience has become a prominent concept within educational research and practice, often conceptualized as a process that enables educators to adapt to adversity, sustain well-being, and remain engaged in their professional roles (Beltman & Mansfield, 2018; Gibbs & Miller, 2014; Gu, 2014; Kutsyuruba et al., 2019). As educational systems navigate rapid change, growing complexity, and mounting expectations, educator well-being has become an increasingly urgent concern. Across diverse educational contexts, educators are experiencing elevated levels of stress, burnout, emotional exhaustion, and attrition (Cadena-Povea et al., 2025; Mijakoski et al., 2022; Sabagh et al., 2018; Turner & Garvis, 2023; Weiland, 2021). Consequently, a growing body of scholarship has sought to better understand the factors that promote educator resilience, coping, and well-being (e.g., Cook et al., 2016; Hill-Berry & Burris-Melville, 2025; Mieres-Chacaltana et al., 2025; Salvo-Garrido et al., 2025). Despite this interest, comparatively less attention has been paid to how contemporary patterns of uncertainty may be reshaping the conditions under which educator resilience is enacted, supported, and sustained.

Although resilience and well-being are closely related, they represent distinct yet complementary constructs (Hascher et al., 2021). In this essay, educator well-being is informed by the World Health Organization’s (2025) conceptualization of mental health as a state of well-being in which individuals are able to cope with the normal stresses of life, work productively, and contribute meaningfully to their communities. Resilience, meanwhile, refers to the dynamic processes through which individuals and systems navigate to, access, and negotiate the resources necessary to sustain well-being in contexts of adversity and change (Ungar, 2012, 2021). While resilience can contribute to well-being, the relation is reciprocal; experiences of well-being may also strengthen educators’ capacities to respond adaptively to future challenges. Consistent with Hascher et al.’s (2021) integrative model, teacher well-being and resilience are conceptualized as distinct yet interconnected processes that emerge through ongoing interactions between educators and the personal, relational, organizational, and contextual resources available within their environments. This social–ecological perspective provides an important foundation for considering how contemporary patterns of uncertainty may influence not only educator well-being but also the conditions under which resilience is enacted and sustained.

Educators work within environments characterized by multiple and often overlapping sources of uncertainty. Uncertainty has been conceptualized as an individual’s perceived inability to accurately predict future events or determine appropriate courses of actions because information is incomplete, ambiguous, or continually evolving (Milliken, 1987). Unlike risk, where probabilities and potential outcomes can be estimated and managed, uncertainty is characterized by indeterminacy regarding what is occurring, what it means, and how one should respond (Capolla, 2024). Within education, uncertainty is recognized not as an exception but as an inherent feature of teaching and learning. Educators routinely encounter disciplinary, curricular, pedagogical, and contextual uncertainty that require ongoing professional judgement and adaptation (Bähr et al., 2026; Bonnet & Glazier, 2025). Importantly, uncertainty is distinct from challenge. While educators frequently encounter challenges that are demanding yet relatively predictable, uncertainty arises when future conditions, expectations, and appropriate responses remain ambiguous or difficult to anticipate. Challenges and uncertainty often co-exist; it is this ambiguity and indeterminacy—not simply the presence of difficulty—that distinguishes uncertainty as a concept.

Emerging technologies such as generative artificial intelligence (Roble, 2024; Kumari et al., 2026; Acevedo, 2025; Zheng, 2025; Nieminen, 2026; Al-Zahrani, 2024; Zootzky & Pfeiffer, 2024), political and social polarization (Zembylas, 2025; Fay & Levinson, 2017; Ruggles, 2025; Glover, 2026; Brandt & Lopes Cardozo, 2023; Marchais et al., 2024; Burridge & Buchanan, 2022; Stitzlein, 2024; Standish, 2024; Muller, 2023; Harvey & Farnoud, 2026), climate-related concerns (Kozyreva & Hertwig, 2021; Dunlop et al., 2021; Loisel, 2022; Kreisel, 2024; Olsen, 2025; White et al., 2024), shifting policy landscapes (Zhao & Zhong, 2025; Stevens, 2022; Rowe, 2025; Parcerisa et al., 2022.; Glassow, 2024), workforce pressures (Longmuir & McKay, 2024; Creagh et al., 2025; Collie, 2022), and the lasting effects of public health disruptions each introduce new challenges and ambiguities for educational practice (Baker et al., 2021; Davis & Park, 2025; Camp et al., 2025; Klusmann et al., 2022). Despite growing interest in educator resilience, less attention has been paid to how these sources of uncertainty converge, interact, and place competing demands on the adaptive resources that support educator well-being and resilience. As these uncertainties compete for finite adaptive resources including time, emotional energy, professional judgment, collegial support, leadership, and organizational capacity, they may influence educators’ abilities to sustain well-being and resilience over time. Existing approaches may underestimate the complexity of the conditions in which resilience is enacted. The contribution of this essay is not simply recognizing that educators face multiple uncertainties nor to catalogue all contemporary challenges facing educators. Rather, we seek to explore how the concept of stacked uncertainty may contribute to current conversations about educator well-being and resilience. We propose the concept of *stacked uncertainties* as a lens for understanding how multiple forms of uncertainty accumulate, interact, and compete for adaptive resources that support educator well-being and resilience.

## 2. Understanding Uncertainty in Contemporary Education

Contemporary education is characterized by uncertainty (Bonnet & Glazier, 2025). Educators are navigating rapid technological innovation, evolving societal expectations, political polarization, climate-related effects and concerns, workforce pressures, shifting policy environments, and the lingering effects of public health disruptions. Although each of these developments has generated its own body of scholarship, they are often described as discrete phenomena. Less attention has been paid to how multiple uncertainties may accumulate, interact, and potentially amplify one another and the implications this may have for educator well-being and resilience.

Existing scholarship has categorized uncertainty according to educational processes, including disciplinary, curricular, pedagogical, and contextual forms of uncertainty (Bähr et al., 2026; Bonnet & Glazier, 2025). The present essay takes a complementary perspective. Rather than organizing uncertainty according to educational processes, we organize the literature according to broader ecological domains within which educators work. This structure is intended as an organizing heuristic rather than an exhaustive list. Our purpose is to examine how uncertainty arising across multiple ecological systems converge within educators’ professional lives and to explore the implications of these intersecting uncertainties for educator well-being and resilience. Viewed through this ecological lens, the challenge facing educators is not any single source, but the simultaneous navigation of multiple and overlapping ambiguities across interconnected systems. The following sections synthesize uncertainty across four ecological domains before introducing stacked uncertainties as a conceptual lens for understanding how these domains converge within educators’ professional lives.

### 2.1. Technological Uncertainty

Technological change represents one of the most visible domains of uncertainty in contemporary education. The rapid emergence of generative artificial intelligence (genAI) and other digital technologies has introduced uncertainty regarding assessment, academic integrity, privacy, bias, teacher roles, and the competencies students will require in an increasingly automated world. More fundamentally, AI challenges long-standing assumptions about knowledge, expertise, authorship, and the purposes of education itself. If information can be generated, summarized, and evaluated by increasingly sophisticated technologies, educators are confronted with difficult questions regarding what students should learn, how learning should be demonstrated, and which forms of human expertise remain most valuable. While educational technologies have long influenced teaching and learning, recent advances in genAI have introduced uncertainty regarding not only how educators teach, but also what knowledge, skills, dispositions, and forms of judgment should be prioritized within educational systems. As technologies evolve more rapidly than educational policies, ethical guidelines, and best practices, educators are increasingly required to make decisions amid uncertainty regarding future expectations, appropriate educational responses, and evolving professional roles.

The integration of AI in education highlights both opportunities and challenges. While genAI may enhance efficiency, personalization, and access to information, concerns have also emerged regarding data privacy, algorithmic bias, transparency, unequal access to technology, diminished opportunities for critical thinking, and the potential erosion of human connection within educational relationships (Al-Zahrani, 2024). At the same time, educators are increasingly expected to develop AI literacy, model ethical technology use, and support students in navigating rapidly changing digital environments (Zootzky & Pfeiffer, 2024). These shifting expectations create uncertainty regarding professional roles, responsibilities, and future educational practice.

Beyond genAI specifically, technological change contributes to broader uncertainty regarding the nature of knowledge and teaching itself. Educational scholars have argued that contemporary educators must continuously engage in reflective and adaptive practice as educational contexts evolve (Dlouhy-Nelson et al., 2023). Similarly, calls for transformative and holistic pedagogies emphasize the need for approaches that cultivate critical thinking, adaptability, and whole-person development in response to changing societal conditions (Cappiali, 2023). Collectively, these developments suggest that technological uncertainty extends beyond the adoption of new tools and platforms to encompass deeper questions regarding educational purpose, professional identity, and the future of teaching and learning.

Technological uncertainty does not occur in isolation but is shaped by broader social, organizational, and political contexts that influence how technologies are interpreted, implemented, and regulated. Technological uncertainty represents one example of broader ecological systems that generates ambiguity for educators and similar patterns are evident within social and political contexts.

### 2.2. Social and Political Uncertainty

While technological uncertainty has introduced ambiguity regarding educational practice, professional roles, and the future of teaching and learning, these developments do not occur in isolation. Educators increasingly navigate not only technological uncertainty, but also uncertainty arising from shifting societal expectations, political polarization, public scrutiny, and ongoing debates about what knowledge, values, and priorities should guide educational practice.

Social and political uncertainty emerges when educators are required to navigate competing values, contested knowledge, and shifting societal expectations within increasingly polarized environments. As with other forms of uncertainty, educators differ in how they perceive and respond to ambiguity. For example, contemporary uncertainty research from the University of Wisconsin-Madison Addiction Research Centre (2026) suggests that individuals differ not only in their tolerance for ambiguity, but also in the extent to which they perceive uncertainty as threatening, unfair, or disruptive to desired outcomes. Within educational settings, these broader societal dynamics often manifest through debates about curriculum, inclusion, identity, historical narratives, and the role of schools in addressing complex social issues. Educators are often required to make professional decisions within contexts where expectations, values, and acceptable practices may be contested by multiple stakeholders (Burridge & Buchanan, 2022).

Educators employ a range of strategies to navigate socio-political tensions within their classrooms. For example, Stitzlein (2024) identified approaches that included reasoning, engaging, communicating, and maintaining a democratic frame. These findings highlight a central source of uncertainty for educators: determining how to respond to complex social and political issues while maintaining alignment with professional responsibilities, school policies, and community expectations. Such decisions often require educators to continuously negotiate competing demands without clear or universally accepted solutions.

Social and political uncertainty is further amplified by growing public scrutiny of educational systems and practices. Schools are increasingly subject to evaluation through accountability measures, public reporting systems, social media discourse, and broader political debates regarding educational effectiveness (Cochran-Smith, 2021). While scrutiny can promote transparency and improvement, it may also create uncertainty regarding how educational decisions will be interpreted and evaluated by various audiences. Questions about curriculum, pedagogy, and student outcomes frequently extend beyond professional educational discussions and become embedded within wider social and political conversations (Standish, 2024). As educational issues become intertwined with broader societal debates, the boundaries between professional, political, and community concerns become less distinct, contributing to greater complexity in educators’ work and creating additional demands on their emotional, relational, and professional resources.

Curriculum debates provide a particularly salient example of social and political uncertainty. Decisions regarding what knowledge should be taught, whose perspectives should be represented, and how complex issues should be addressed often reflect broader societal disagreements about identity, inclusion, equity, and power. Muller (2023) describes tensions between sociological approaches to curriculum, which emphasize broader social and theoretical considerations, and more discipline-focused approaches that prioritize subject-specific knowledge. Similarly, Harvey and Farnoud’s (2026) conceptualization of the “Onion of Oppression” highlights how questions of identity, hierarchy, privilege, and oppression may become sites of both learning and conflict within educational contexts. While engagement with these issues is essential to contemporary education, they can also contribute to uncertainty when educators encounter competing narratives, misinformation, and polarized public discourse.

Debates regarding curriculum, inclusion, misinformation, and educational purpose frequently intersect with technological developments, policy reforms, organizational pressures, and broader societal transformations. Social and political uncertainty is rarely experienced independently of other ecological domains.

### 2.3. Ecological Uncertainty

While technological and socio-political uncertainties often emerge through changing practices, policies, and societal expectations, ecological uncertainty is distinguished by ambiguity surrounding future environmental conditions. The climate crisis and the broader processes of ecological degradation present complex and evolving challenges whose consequences are often difficult to predict, creating uncertainty regarding environmental, social, economic, and educational futures (Cutter, 2020). Kozyreva and Hertwig (2021) distinguish between aleatory uncertainty, arising from inherently unpredictable events, and epistemic uncertainty, stemming from limitations in knowledge and understanding. Within educational contexts, both forms of uncertainty are relevant as educators and students grapple with the realities of climate crisis while attempting to make sense of uncertain futures. Educators must respond not only to unpredictable environmental events, but also to evolving scientific knowledge and the inherent limits of forecasting future ecological and societal conditions.

In response to these challenges, the importance of meaningful climate education extends beyond the transmission of information. Dunlop et al. (2021) describe the importance of participation, policy, and pedagogy in supporting educational responses to the climate crisis, arguing that young people should be engaged not only in learning about climate change but also in critically imagining and responding to future possibilities. These perspectives position educators as important facilitators of dialogue, reflection, and action in relation to complex ecological challenges. However, this role is accompanied by uncertainty as educators navigate shifting public discourse and competing perspectives regarding climate action and responsibility.

Beyond structural and pedagogical considerations, educators must also contend with the emotional dimensions of ecological uncertainty. Emerging scholarship has documented experiences of climate grief, eco-anxiety, and anticipatory loss associated with the climate crisis and ongoing ecological degradation (Galway & Field, 2023; Hickman et al., 2021). Loisel (2022) describes eco-grief as encompassing feelings of stress, fear, worry, and loss associated with anticipated or ongoing environmental disruption. Within educational settings, these emotions may be experienced not only by students but also by educators themselves. Kreisel (2024) documented feelings of demoralization and disengagement among students who appeared overwhelmed by the scale and complexity of the climate crisis.

Ecological uncertainty involves not only cognitive but also emotional dimensions, requiring climate education for supporting emotional engagement alongside knowledge development. Olsen (2025) argues for approaches that connect ecological concerns to personal values, collective meaning-making, and opportunities for action. Such approaches may help transform feelings of helplessness into engagement and agency while creating space for reflection and dialogue. Yet this work also requires educators to navigate uncertainty regarding how to foster hope and action without minimizing the realities of the climate crisis, ecological degradation, and their associated experiences of loss, disruption, and environmental change (Lawrence et al., 2022).

Ecological uncertainty also intersects with broader technological, social, political, and organizational changes. White et al. (2024), drawing on educators’ experiences during both the climate crisis and the COVID-19 pandemic, highlighted how uncertainty, discomfort, and risk often coexist within contemporary educational work. Educators are increasingly asked to prepare students for futures shaped by climate instability and ecological degradation while simultaneously navigating their own uncertainty, grief, and concerns about those same futures. Ecological uncertainty therefore encompasses both a practical and emotional dimension of contemporary educational work.

### 2.4. Organizational Uncertainty

Educational organizations are the primary setting through which broader technological, socio-political, and ecological uncertainties are experienced by educators. Schools, districts, and educational institutions provide the organizational contexts in which policy reforms, technological innovations, accountability measures, workforce challenges, and resource constraints are implemented and enacted. Organizational uncertainty represents an important dimension of educational work, shaping how educators experience and respond to broader systems of change.

One prominent domain of organizational uncertainty is the growing challenge of educator recruitment and retention. The United Nations Educational, Scientific and Cultural Organization and International Task Force on Teachers for Education 2030 (2023) estimates a global shortage of 44 million primary and secondary educators by 2030, with factors such as workload, overcrowded classrooms, and inadequate compensation contributing to workforce attrition. In response, educational systems continue to introduce policies and initiatives aimed at recruitment, retention, and organizational improvement. However, Zhao and Zhong (2025) caution that system-level reforms do not necessarily result in meaningful cultural or philosophical change within schools. They further argue that efforts to reduce educator burden may be more effective when implemented at smaller scales where innovations can be adapted to local contexts and needs. These workforce pressures create uncertainty not only about staffing, but also workload, role expectations, collegial support, and the long-term sustainability of educational systems.

Educators and educational leaders increasingly operate within conditions of financial uncertainty arising from unpredictable economic conditions, such as fluctuating funding levels, inflation, and broader financial instability, that influence educational resources, policy priorities, and teachers’ working conditions (Jackson et al., 2021; Dizon-Ross et al., 2019). Stevens (2022) documented the additional time and effort teachers invest in securing supplementary resources, including fundraising initiatives and external funding sources. Similarly, Rowe (2025) found that school leaders frequently described needs-based funding models as coercive, exploitative, and insufficient for addressing organizational and student needs. These findings suggest that financial pressures extend beyond budgetary concerns and may influence educators’ capacities to access resources, implement programs, and support student well-being. As a result, uncertainty regarding available resources becomes intertwined with broader concerns related to workload, organizational capacity, and educational quality.

Organizational uncertainty is further intensified through accountability systems that regulate and evaluate educational practice. Performance-based accountability frameworks increasingly emphasize measurable outcomes and student achievement indicators as markers of educational effectiveness (Parcerisa et al., 2022). While accountability systems may support improvement and transparency, they can also create uncertainty regarding professional autonomy, decision-making, and expectations for success. Glassow (2024), for example, found that performance-based accountability measures were associated with diminished well-being and increased intentions among experienced educators to seek employment elsewhere. Such findings suggest that accountability pressures may shape not only professional practice but also educators’ experiences of stress, satisfaction, and organizational commitment.

Organizational uncertainty reflects the institutional contexts through which broader technological, socio-political, and ecological changes are experienced by educators. Within schools and educational institutions, technological innovations require implementation, curriculum debates become policy decisions, climate priorities shape educational initiatives, and public health challenges influence workplace practices. As a result, educational organizations often become the point at which multiple uncertainties converge. The following section introduces the concept of stacked uncertainties as a lens for understanding these interactions.

## 3. When Uncertainties Become Stacked

**Figure 1:**
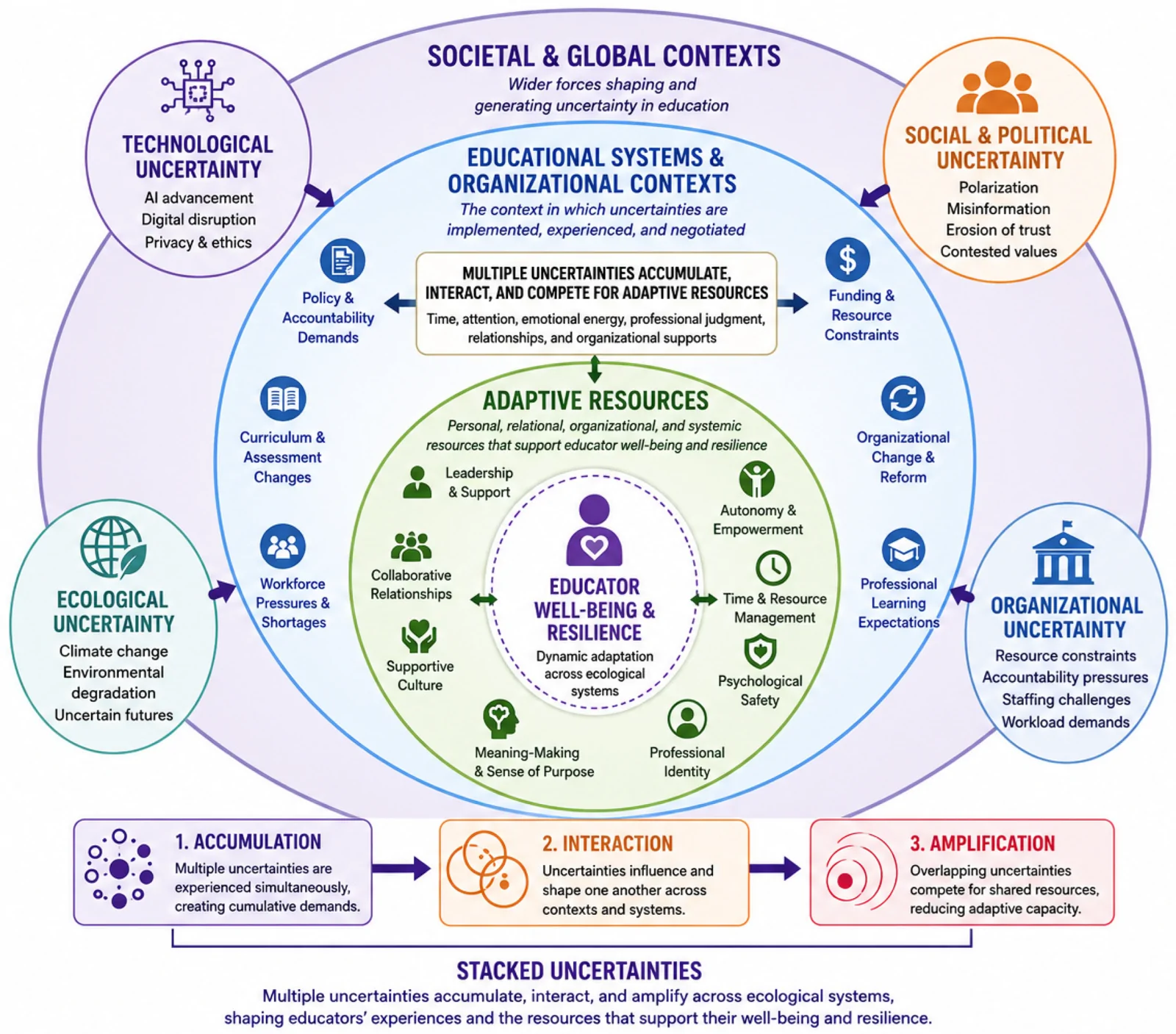
Stacked uncertainties.

The preceding sections described how uncertainties shape contemporary educational contexts and how educators rarely experience them as discrete challenges. Rather, uncertainty increasingly emerges through the simultaneous presence of multiple demands, expectations, and ambiguities operating across interconnected systems. It is this convergence that forms the basis of the concept of stacked uncertainties. The uncertainties discussed in this essay are not intended to represent an exhaustive list of challenges facing educators globally, nor to provide a definitive or reductive categorization of educator uncertainty. Rather, they reflect prominent sources of uncertainty identified within the contemporary educational literature, much of which has emerged from Global North contexts (Bonnet & Glazier, 2025; Capolla, 2024; Gilead & Dishon, 2022). Educational settings may experience additional domains or different forms of uncertainty related to poverty, economic insecurity, violence, conflict, displacement, political instability, public health crises, or other contextual factors. The concept of stacked uncertainties is intended as a conceptual lens for understanding how multiple uncertainties may accumulate, interact, and potentially amplify across ecological systems. Drawing on social–ecological perspectives, the concept recognizes that educators are embedded within nested and interacting contexts that include classrooms, schools, communities, policy environments, technological systems, and broader societal and ecological conditions. Consequently, uncertainty may arise not only from individual events or changes but from the ways in which multiple uncertainties intersect and are experienced concurrently. Importantly, these uncertainties often draw upon the same personal, relational, organizational, and material resources, creating conditions in which demands may exceed available capacities for adaptation and response. Stacked uncertainties emphasize how multiple uncertainties operate simultaneously across ecological systems and compete for the same adaptive resources. Figure 1 illustrates the proposed conceptual lens, depicting how multiple domains of uncertainty converge across ecological systems and influence educator well-being and resilience through the processes of accumulation, interaction and amplification.

### 3.1. Accumulation of Uncertainty

The first process associated with stacked uncertainties is accumulation. Educators may simultaneously encounter technological uncertainty related to AI and evolving teaching practices, social and political uncertainty arising from curriculum debates and public scrutiny, ecological uncertainty associated with the climate crisis, and organizational uncertainty linked to staffing shortages, accountability pressures, and resource constraints. While each uncertainty may present significant demands, their simultaneous presence can create a sense of cumulative pressure and complexity.

For example, a secondary school teacher may be redesigning assessments in response to genAI while simultaneously grappling with ethical questions related to academic integrity, student privacy, bias, authorship, and the appropriate role of AI in teaching and learning. These decisions may also require educators to critically consider how emerging technologies are reshaping knowledge production, work, and the purposes of education within increasingly automated and market-driven societies. At the same time, they may be teaching environmental sustainability in ways that evoke student anxiety, grief, or difficult conversations about uncertain futures, while also navigating parental concerns regarding curriculum content, responding to increasing accountability expectations related to student achievement, and managing larger class sizes resulting from organizational demands or staffing shortages. Educators may be able to devote substantial attention and resources to any one of these challenges, drawing upon professional knowledge, coping strategies, adaptive resources, and available supports, but the simultaneous occurrence creates a complex web of demands requiring ongoing decision-making, emotional labour, adaptation, and professional judgment. The challenge lies not in responding to a single source of uncertainty but in navigating multiple and interacting uncertainties that may compete for the same personal, relational, organizational, and material resources. These demands do not occur sequentially but often unfold simultaneously within the same classroom, school, and academic year. The accumulation of uncertainty may therefore increase both the complexity of educator work and the resources required to respond effectively.

### 3.2. Interaction Among Uncertainties

A second process associated with stacked uncertainties is interaction. Uncertainties do not simply coexist; they frequently influence and shape one another. Technological developments may intensify social and political debates regarding misinformation, privacy, equity, and educational purpose. Climate crisis may become entangled with political polarization, economic priorities, and public discourse. Organizational policies may shape how educators are expected to respond to technological innovations, curriculum controversies, or public health concerns. In turn, these responses may generate additional demands on educators’ time, attention, and professional judgment.

From an ecological perspective, these interactions create conditions in which the boundaries between different forms of uncertainty become increasingly blurred. For educators, uncertainty is often experienced as a network of interconnected challenges rather than a series of isolated problems. Understanding these interactions is important because the effects of uncertainty may be shaped not only by its source but also by the broader context in which it occurs. A policy initiative intended to improve accountability, for example, may interact with staffing shortages, technological change, and competing curricular demands in ways that create new and unanticipated pressures for educators. Similarly, climate-related discussions may intersect with political debates, community values, and questions regarding the role of education in responding to societal challenges. Such interactions suggest that educators are often navigating multiple layers of uncertainty simultaneously rather than responding to isolated challenges.

### 3.3. Amplification of Uncertainty

A third process associated with stacked uncertainties is amplification. Amplification occurs when overlapping uncertainties place concurrent demands on shared adaptive resources, creating conditions in which educators may experience reduced capacity to respond effectively despite continued efforts to adapt. Time, emotional energy, professional judgment, collegial support, leadership capacity, and organizational resources are finite. As multiple uncertainties emerge simultaneously, they may compete for these shared resources, reducing educators’ capacity to respond effectively to any one challenge.

Importantly, not all uncertainties exert equal influence, nor do all combinations of uncertainties produce the same effects. Certain forms of uncertainty may be relatively manageable when adequate supports are available, whereas others may become particularly challenging when combined with existing stressors or resource limitations. For example, adapting to the opportunities and challenges associated with genAI may be experienced differently in a well-resourced school with supportive leadership, professional learning opportunities, and access to technological resources than in a context already facing staffing shortages, accountability pressures, and competing organizational demands. Similarly, teaching about the climate crisis may have different implications depending on educators’ access to collaborative networks, curricular supports, and opportunities for collective reflection and action.

The effects of stacked uncertainties are also shaped by broader social and structural conditions. Existing inequities related to poverty, economic insecurity, resource scarcity, violence, and other forms of structural disadvantage may further amplify the effects of uncertainty by reducing access to the personal, relational, organizational, and material resources that support adaptation and well-being. In Canada, these inequities are further shaped by the ongoing impacts of colonialism and continuing efforts toward reconciliation, which have prompted important reflection regarding Indigenous knowledges, educational purposes, curriculum, and institutional change (Dlouhy-Nelson et al., 2023). Consequently, both exposure to uncertainty and access to adaptive resources may be unevenly distributed across educational contexts.

Amplification occurs when multiple uncertainties converge within contexts where adaptive resources are finite or unevenly distributed, reducing educators’ capacities to respond effectively despite continued efforts to adapt. Together, the processes of accumulation, interaction, and amplification illustrate that uncertainty is experienced as a dynamic and interconnected ecological phenomenon rather than as a series of isolated stressors. From this perspective, educator well-being and resilience are shaped not only by individual coping capacities but also by the broader ecological conditions in which multiple uncertainties converge and adaptive resources are mobilized.

## 4. Implications for Educator Resilience and Well-Being

Contemporary resilience scholarship increasingly conceptualizes resilience as a dynamic and contextually situated process that emerges through interactions between individuals and their environments rather than as a fixed personal characteristic. From a social–ecological perspective, resilience is shaped by access to resources, opportunities, relationships, and systems that support adaptation in the face of adversity and change (Ungar, 2012, 2021). This perspective is particularly relevant when considering stacked uncertainties, as the challenges facing educators emerge across multiple ecological systems, including individual and broader societal contexts. Educator well-being may therefore be shaped not only by the presence of multiple demands but also by the extent to which those demands compete for the same adaptive resources. As uncertainties accumulate, interact, and amplify, educators increasingly rely upon personal, relational, organizational, and systemic resources to navigate ongoing change. Consequently, resilience may be understood less as an individual capacity to withstand adversity and more as a dynamic process through which resources are accessed, negotiated, mobilized, and sustained across ecological systems. The implications of stacked uncertainties can be understood across three interconnected levels: individual, relational, and organizational.

At the individual level, meaning-making and professional identity may represent important adaptive resources (Flores, 2018; Kamrath & Tracy, 2026). Educators are increasingly required to navigate changing expectations regarding knowledge, technology, curriculum, inclusion, and the broader purposes of education. In contexts characterized by uncertainty, opportunities to interpret, reflect upon, and make sense of change may help sustain coherence, purpose, and direction within professional practice. Kamrath and Tracy (2026) describe how resilience may emerge through *affirmative sensemaking*, whereby educators reinterpret previous experiences of challenge as sources of knowledge, confidence, and adaptive capacity that inform responses to future uncertainty. Rather than simply recovering from disruption, educators may construct narratives of competence, growth, and possibility that sustain professional purpose amid ongoing uncertainty. Professional identity may similarly function as an important identity anchor during periods of disruption (Flores, 2018). As educational expectations evolve, maintaining a coherent sense of purpose while adapting professional practice may enable educators to integrate change without experiencing every uncertainty as a threat to who they are as educators (Hascher et al., 2021; Kamrath & Tracy, 2026).

At the relational level, resilience is not only supported through relationships but also constructed through them. Collegial relationships, professional collaboration, and a sense of community and belonging provide opportunities not only for emotional support but also for shared interpretation of uncertainty (Gu, 2014; Joseph et al., 2022). Through dialogue, collaborative reflection, and collective problem-solving, educators may develop what Kamrath and Tracy (2026) describe as *critical co-reflexivity*—a collaborative process through which individual experiences are transformed into shared understanding and collective capacity. As educators share stories of navigating uncertainty, individual experiences become collective resources that inform future responses, normalize challenge, and strengthen professional communities. These relational processes may enable educators to pool knowledge, coordinate adaptive responses, and sustain well-being when uncertainties exceed the capacity of any one individual to respond independently.

At the organizational level, leadership, culture, and working conditions become increasingly important under conditions of stacked uncertainty (Theiss et al., 2025). Supportive leadership may foster psychological safety, transparent communication, flexibility, and shared decision-making, creating conditions in which educators feel able to acknowledge uncertainty, seek support, and collectively explore adaptive responses (Acton, 2025; Weiner et al., 2021). Organizational cultures that encourage reflection, collaboration, and professional learning may enhance educator access to adaptive resources, whereas inconsistent communication, limited trust, competing priorities, or chronic resource constraints may amplify uncertainty by reducing opportunities for collective problem-solving. From a social–ecological perspective, resilience depends not only on the educators’ capacities but also on the extent to which educational systems create environments that enable those capacities to flourish.

The concept of stacked uncertainties highlights the importance of collective resilience. Because many of the uncertainties facing educators originate within broader technological, political, ecological, organizational, and economic systems, responses focused solely on individual coping are unlikely to be sufficient. Rather, resilience may be understood as a collective capacity that emerges through relationships, organizational cultures, leadership practices, and professional communities that support adaptation across multiple levels of the educational system (Ebersohn, 2020; Norris et al., 2008; Pi Ferrer et al., 2025). As educators collectively interpret uncertainty, share knowledge, and develop coordinated responses, resilience becomes embedded within the practices and cultures of educational organizations rather than residing solely within individuals. From this perspective, supporting educator well-being requires strengthening the relational and organizational conditions that enable educators to navigate uncertainty together.

Implications for future research, educational practice, leadership, and policy focus on how multiple uncertainties shape educator well-being and resilience by influencing access to adaptive resources across ecological systems. Future research may benefit from exploring how multiple uncertainties are experienced simultaneously and how adaptive resources influence educators’ capacities to navigate ongoing change. Longitudinal and mixed-methods research may be particularly valuable for understanding how stacked uncertainties evolve over time, while new measures may help capture both educator experiences of uncertainty and the resources available to support adaptation. For educational practice, this perspective suggests that supporting educator well-being requires more than strengthening individual coping strategies. Opportunities for collaborative reflection, mentoring, communities of practice, and professional dialogue may enable educators to collectively interpret uncertainty, share adaptive strategies, and develop shared meaning. Processes such as affirmative sensemaking and critical co-reflexivity may further strengthen collective adaptive capacity by transforming individual experiences into shared knowledge (Hascher et al., 2021; Kamrath & Tracy, 2026). Educational leaders and policymakers also play an important role in shaping the conditions that support resilience. Although uncertainty cannot always be reduced, leaders can strengthen educator access to adaptive resources by fostering psychological safety, organizational trust, collaborative decision-making, professional learning, and coherent communication (Glassow, 2024; Hascher et al., 2021; Parcerisa et al., 2022; Ungar, 2021; Zhao & Zhong, 2025). Ultimately, the stacked uncertainties conceptual lens shifts attention from helping individual educators cope with isolated challenges toward strengthening the collective and ecological conditions that enable educational systems to adapt and educator well-being and resilience amid ongoing complexity and change.

## 5. Conclusions

Educators are increasingly working within environments characterized not simply by rapid change, but by multiple and overlapping forms of uncertainty. While technological innovation, political polarization, ecological disruption, organizational pressures, workforce challenges, and public health concerns are often examined independently, this essay has argued that they are frequently experienced simultaneously within educators’ professional lives. The concept of stacked uncertainties provides a conceptual lens for understanding how these uncertainties accumulate, interact, and amplify by competing for the adaptive resources that support educator well-being and resilience.

As a conceptual paper, the proposed lens has several limitations. The four domains of uncertainty are presented as an organizing heuristic rather than an exhaustive taxonomy of educator uncertainty, and their relative importance is likely to vary across educational settings, disciplines, professional roles, and geographic contexts. Similarly, the immediacy and influence of different uncertainty domains may vary, with some experienced directly through educators’ professional practice and others exerting more indirect or context-dependent effects. Future research could examine whether distinct configurations, clusters, or potentially nested forms of uncertainty emerge across educational contexts, and whether particular combinations have different implications for educator well-being and resilience. Advancing the concept of stacked uncertainties will require sustained programs of research that refine the proposed conceptual lens, identify the adaptive resources that are most protective, and evaluate strategies for strengthening educator well-being and resilience amid multiple, overlapping uncertainties.

The contribution of this conceptual lens is not simply recognizing that educators face multiple challenges. Rather, it highlights that contemporary educational work is increasingly characterized by overlapping uncertainties that reshape the conditions under which educator well-being and resilience are enacted by placing simultaneous demands on finite adaptive resources across interconnected ecological systems. From this perspective, supporting educator resilience requires more than helping individuals cope with isolated stressors; it also requires strengthening the relational, organizational, and systemic conditions that enable educators to adapt, collaborate, and sustain well-being over time. Although uncertainty will remain an enduring feature of educational work, educational systems can cultivate the conditions that enable educators to navigate complexity collectively. By reframing uncertainty as an ecological phenomenon rather than a collection of isolated challenges, the concept of stacked uncertainties offers a perspective for future research and contributes to ongoing efforts to understand and support educator well-being and resilience in an era of rapid and ongoing change.

## Data Availability

No new data were created or analysed in this study. Data sharing is not applicable to this article.

## Abbreviations

The following abbreviations are used in this manuscript:

GenAI	Generative Artificial Intelligence
